# Right Care for the Right Patient Each and Every Time

**DOI:** 10.7759/cureus.492

**Published:** 2016-02-12

**Authors:** Amar Basavatia, Jose Fret, Alex Lukaj, Hsiang Kuo, Ravindra Yaparpalvi, Wolfgang A Tome, Shalom Kalnicki

**Affiliations:** 1 Department of Radiation Oncology, Montefiore Medical Center; 2 Montefiore Information Technologies, Montefiore Medical Center; 3 Montefiore Medical Center, Albert Einstein Medical School

**Keywords:** patient safety, biometrics, risk mitigation, patient satisfaction

## Abstract

**Purpose:**

To implement a biometric patient identification system in the field of radiation oncology.

**Materials and Methods:**

A biometric system using palm vein scanning technology has been implemented to ensure the delivery of treatment to the correct patient each and every time. By interfacing a palm vein biometrics system (PVBS) (PatientSecure®, Imprivata, Lexington, Massachusetts) with the radiation oncology patient management system (ROPMS) (ARIA®, Varian Medical Systems, Palo Alto, California) one can integrate patient check-in at the front desk and identify and open the correct treatment record of the patient at the point of care prior to the initiation of the radiation therapy treatment.

**Results:**

The learning time for the use of the software and palm scanner was extremely short. The staff at the front desk and treatment machines learned the procedures to use, clean, and care for the device in one hour’s time. The first key to the success of the system is to have a policy and procedure in place; such a procedure was created and put in place in the department from the first day. The second key to the success is the actual hand placement on the scanner. Learning the proper placement and gently reminding patients from time to time was found to be efficient and to work well.

**Conclusion:**

The use of a biometric patient identification system employing palm vein technology allows one to ensure that the right care is delivered to the right patient each and every time. Documentation through the PVBS database now exists to show that this has taken place.

## Introduction

The overall goal of patient safety measures in radiation therapy is to ensure that the right treatment is delivered to the right patient and the correct anatomical site at every treatment visit. As part of our continuous quality management program [1], we sought to further mitigate the risk of patient misidentification at the point of care initiation by strengthening the patient identification process even further through the use of palm vein biometric technology (PVBS). Prior to the implementation of PVBS, our patient identification procedure used the commonly employed patient identification system in radiation oncology consisting of checking the face photo, the patient’s first and last names, and the patient’s date of birth (DOB). Despite these checks, at least one patient was mistreated prior to implementing PVBS. Since our clinic can provide care simultaneously for up to 30 patients, who may have the same or very similar first and last names, the use of the patient’s first and last name was identified as a potential point of failure. Adding palm vein scanning as an additional layer of safety made the patient identification process robust and more personal at the point of care initiation. Palm vein scanning utilizes an infrared beam on the palm of one’s hand to read the unique vein patterns of the individual [2]. The vascular structure of veins in the palm of the hand are 100 times more unique than fingerprints and are much less cumbersome than iris/retinal scans of the patient. In this article, we report how this technology can be integrated into the workflow and into the clinical patient management software to augment and strengthen the patient identification process at the point of care initiation.

## Materials and methods

After mapping out our clinical workflow, two areas were identified for integration—the front desk and the treatment machine. To map out the workflow, we followed the patient through the department to see the points of contact with individuals and technology to ascertain where and how a misidentification can take place. We were also interested to determine where the process could be automated or where technology could be added to help mitigate the risk of patient misidentification. The crucial area identified was the point of care at the treatment machine. At the treatment machine, prior to care initiation, there is a manual process of selecting the patient from a list of patients on treatment for the day. The list, or queue for the day, can contain patients with the same first and last names. Our database has over 20,000 records of patients with many instances of patients with the same or very similar first and last names. More often than not, two such patients are under treatment at the same treatment machine increasing the risk of patient misidentification using a manual patient identification methodology. There may be up to 180 patients on treatment on any given day. This is where a palm vein biometrics system (PVBS) (PatientSecure®, Imprivata, Lexington, Massachusetts) can add an extra layer of safety. 

**Figure 1:**
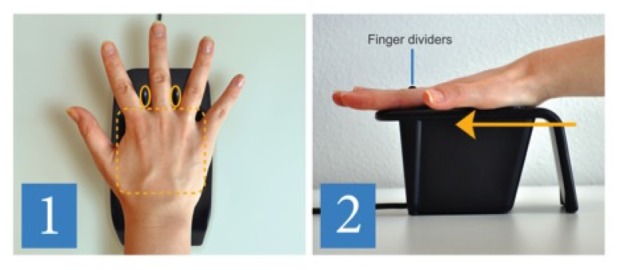
A hand placement guide next to each palm vein scanner depicting the correct hand placement.

At the front desk, the staff or registrar has the patient registration module of the ROPMS (ARIA®, Varian Medical Systems, Palo Alto, California) along with other electronic medical records (EMR) applications open at their workstations. Patient tracker views in the ROPMS are customizable and can be integrated with the PVBS. The PVBS utilizes its own server for patient record and palm vein image storage and utilizes the ADT (Admissions, Discharges, Transfers) interface for communication and patient identification in our ROPMS. The patient’s name, DOB, and address are fed into the PVBS application prior to patient identification through the palm vein scanning. Upon a patient’s first arrival to the department, a face photo is taken and entered into the ROPMS. The patient's demographic information is also checked for accuracy and missing information is entered. Then, in the PVBS application, the DOB is entered and a palm vein scan is registered or recorded for the first time. The registration requires two scans of the palm. Instructions are given to the patient for correct palm placement and hand position. The correct technique is to make a “five”—extend all fingers with the fingers spread apart and then to slide the hand onto the reader until the guides stop the hand. The majority of patients have no problem learning or applying this technique within one or two tries. However, some patients have difficulty either understanding or opening their fingers due to arthritis and require multiple tries before mastering the technique to allow reliable and reproducible identification. To help with the technique, an illustrated hand guide placement picture has been placed next to the reader (Figure 1). 

**Figure 2:**
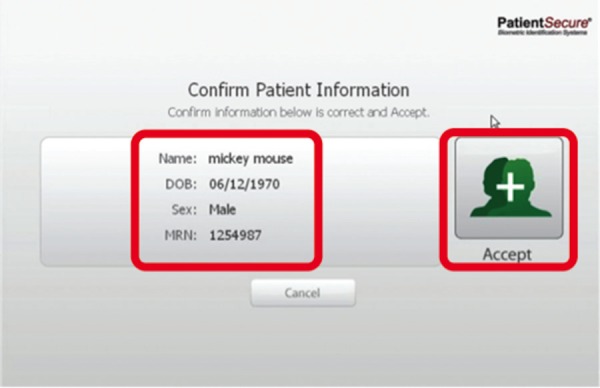
Confirmation of the correct patient before scanning of the palm at the front desk.

**Figure 3:**
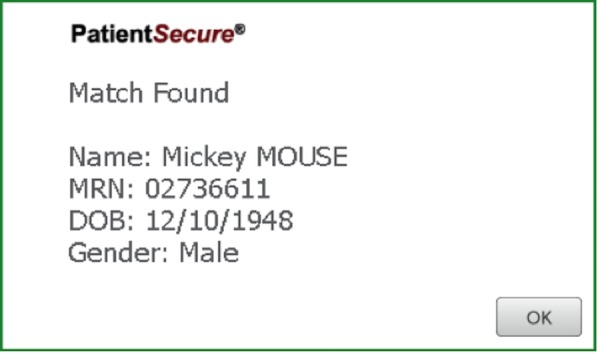
A successful match of palm vein scanning of the patient at the front desk.

**Figure 4:**
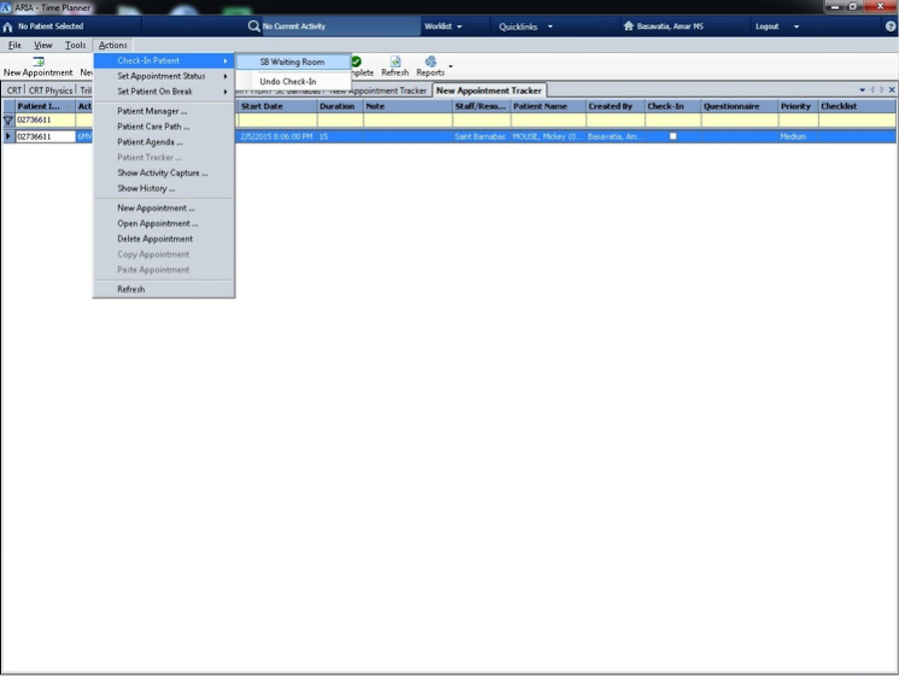
Check-in of the patient into the ROPMS after a successful scan.

For subsequent visits by the patient, the registrar clicks on the patient tracker screen in ROPMS and then onto the PVBS screen. The registrar then verifies the DOB and scans the patient’s palm (Figure 2). After a successful match is made, a confirmation screen (Figure 3), is shown and then a handoff from the PVBS to the ROPMS occurs whereby the patient is checked-in to their appointment. A second confirmation screen is shown when the handoff occurs to the ROPMS (Figure 4). The entire check-in process takes a minute or less for many patients. 

The following hardware—a PVBS server, the existing ROPMS servers, an ADT interface, USB Fujitsu Palm Readers [3], and PalmSecure® (Fujitsu, Tampa, Florida)—was attached to workstations to implement our biometric patient identification system. The PVBS software resides only in the workstations where a palm reader is attached. Based on the workflow and location, i.e., front desk or treatment machine, the user is prompted upon first launch to confirm location. This is a necessary step to keep track of matches by location and function. To customize patient tracker views for the front desk registrar in the ROPMS, the view is created with the medical record number (MRN) as the first column to ensure that the proper information passes through between the two systems. For the treatment machines, the patient queue screen was utilized for integration. This screen has all patients on treatment for the day listed. Note that this list could have 30 or more patients on queue with similar or the same patient names receiving similar types of treatments throughout the day. Moreover, similar treatments tend to be delivered on treatment machines that are most suitable for them thus it is imperative to select the right patient each and every time using a system that has high sensitivity and specificity such as the PVBS. The queue has an area for external input, the auxiliary device interface, (ADI). This input allows the PVBS to send a message identifying the correct patient. Once a message is sent, the correct patient record is opened for treatment. The patient MRN is the message passed through the ADI. To date, no near misses nor mistreatments have occured as a result of this implementation.

As with any new technological initiative, tracking its use and accuracy helps determine the value obtained. As a result, automated monthly SQL (Structure Query Language) reports were built and analyzed. These monthly SQL reports included data on additions to the department, positive matches found for patients in the PVBS database, and unsuccessful scans or no matches found for patients in the PVBS database.

## Results

**Figure 5:**
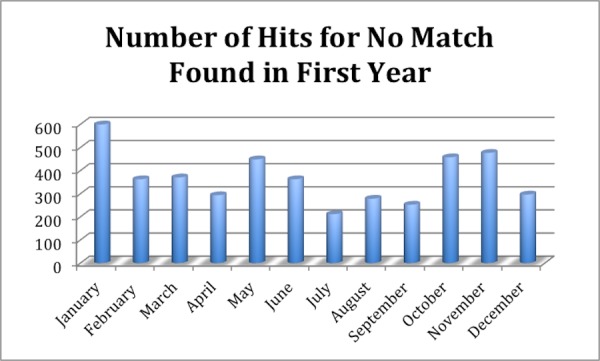
A month-to-month comparison of scans could not be identified. There is an average of three No Matches per patient in any given month. A total of 1434 registered patients with total 4405 No Matches found.

**Figure 6:**
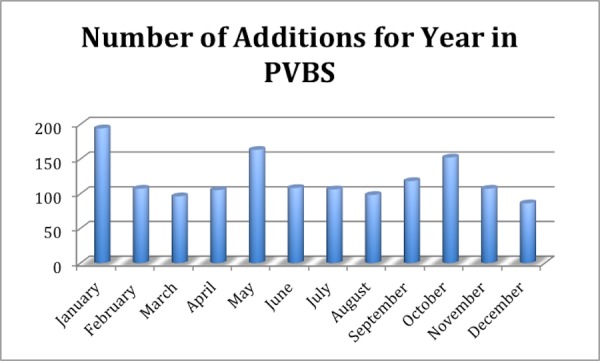
A month-to-month comparison of additions to PVBS. A total of 1434 registered patients.

**Figure 7:**
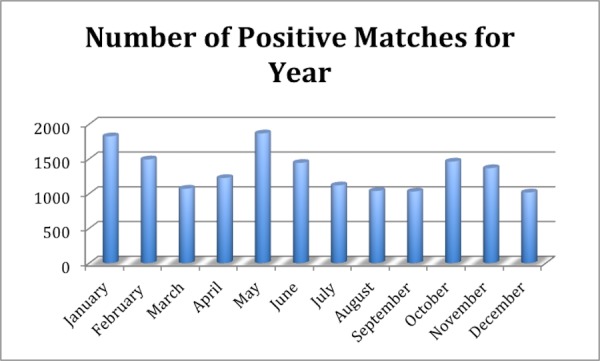
A month-to-month comparison of positive matches to PVBS. A total of 15,946 matches for the year. An average of 11 positive matches per patient. 15,946 matches and 1434 patients registered.

The learning time for the use of the software and palm scanner was extremely short. The staff at the front desk and treatment machines were able to learn all necessary procedures of how to effectively and safely employ the system within one hour. It was found that it is instrumental to have a policy and procedure in place; this was created and put in place from the first day for the successful deployment of this technology throughout the department. Moreover, it was found that proper hand placement on the scanner and having an illustrated hand guide placement picture next to the palm scanner to remind patients of the proper hand placement are key ingredients to reliable and reproducible identification using this technology. Supporting patients in learning the proper placement by using gentle reminders was found to be an extremely effective technique, allowing the majority of patients to master the palm scanning technique within the first few treatment sessions. The success of these efforts is reflected in the low number of unsuccessful matches found in the reports, which is on average three per patient (Figure 5). While this number really never goes to zero it stays at an acceptably low level to allow for successful use of the system in daily clinical use. New patient additions or registrations into the PVBS are shown (Figure 6). As can be seen from Figure 5 and Figure 6, new patient additions (in each month of the year) correlate with the number of no matches found in the PVBS. This indicates that the number of No Matches found is caused by the registrar clicking a button before the patient places their hand on the palm scanner and because it takes a patient a number of tries to learn the correct hand placement on the palm scanner to successfully use the system throughout treatment (Figure 7). 

## Discussion

It has been found that a PVBS can add an additional layer of safety to patient identification and that such a technology can be integrated into the radiation oncology care workflow requiring only minimal modifications of the existing patient care workflow. This integration has been achieved by utilizing the ADT for communication and patient identification in our radiation oncology patient management system (ROPMS). In the hierarchy of safety effectiveness, by the Institute for Safe Medication Practices, [4-7] technology focused automation is at the higher end of the effectiveness chart—second out of six possible places. Coupling the use of PVBS with departmental policies and procedures, which ranks fifth on the list, an effective outcome can be achieved. 

The question one might ask is what if the hand is fractured during the course of treatment? If the right hand is fractured, then of course the left hand can be employed. The right hand record can be deleted in the PVBS system and the left hand palm print can be stored instead. The PVBS has an administration tool that select users can have access to for performing such tasks. In the event that a patient is a double amputee having lost both hands or both hands are fractured during the course of treatment, one has to revert to the traditional manual way of patient identification. Moreover, since both of these events are low probability events, the existence of such a patient in the department does not present an undue safety risk for patient misidentification since any other patient with two intact or one broken hand can still utilize the technology. While the unique palm vein pattern of a patient does not change from birth to death, special workflow procedures are recommended for the use of the PVBS technology with pediatric patients. For patients 16 years of age or younger, it is recommended that the patient be re-enrolled following successful authentication if it has been more than a year since their last visit. While the vein pattern does not change, the size of the hand is growing and that can affect consistent hand placement. The system automatically prompts for the re-enrollment based on patient age, current date of service, and last date of service. 

One way to further enhance the patient experience with PVBS is to add a self check-in kiosk at the front desk. This will allow patients during their daily visits to quickly and accurately self check-in for their daily appointment.

## Conclusions

The use of palm vein technology allows one to ensure that the right care is delivered to the right patient at the point of care prior to care initiation. With the PVBS database, clear documentation exists that the correct treatment was given to the correct patient for each treatment. This documentation could not be done without biometrics. The use of PVBS technology can help in significantly reducing the risk of patient misidentification at the point of care. There are 1225 misadministrations each year in radiation therapy across the United States [8]. The use of PVBS in radiation therapy could substantially reduce this number, though not to zero since factors other than patient misidentification affect this number. Waiting for radiotherapy equipment manufacturers to include all of the fail-safe measures in their products is not always feasible. The integration of third party technology into the workflow and the use of the radiation therapy equipment employing existing and approved interfaces coupled with departmental policies and procedures represents one avenue that can drive down error rates. A culture of safety in the department is necessary for effective outcomes. By automating the matching of the correct treatment to the correct patient each and every time, the risk for injury will be reduced for patients and henceforth the total cost of care.
